# Local Anesthetic Systemic Toxicity Complicating Thyroid Biopsy

**DOI:** 10.7759/cureus.1955

**Published:** 2017-12-16

**Authors:** Jean Liew, James Lundblad, Adam Obley

**Affiliations:** 1 Internal Medicine, University of Washington; 2 Division of Endocrinology, Department of Medicine, Oregon Health and Science University, Portland, Oregon; 3 Department of Medicine, Oregon Health and Science University, Portland, Oregon

**Keywords:** thyroid nodule, thyroid cancer, overtreatment, overdiagnosis, local anesthetic systemic toxicity, lidocaine

## Abstract

The overdiagnosis of thyroid malignancies may be contributing to the increased incidence of these cancers with a relatively stable mortality rate. We present the case of a man with known malignancies, who underwent biopsy of a suspicious thyroid nodule. This procedure was complicated by local anesthetic systemic toxicity (LAST). It is important to address goals of diagnostic testing and treatment with patients, particularly if further evaluation is unlikely to change management or outcomes.

## Introduction

In the United States, the incidence of thyroid cancer has risen from 4.9 to 14.3 per 100,000 since 1975, while the death rate attributable to thyroid cancer has remained stable at 0.5 per 100,000. Most people diagnosed with thyroid cancer in the United States opt to undergo thyroidectomy, sometimes with lymph node dissection, and treatment with radioactive iodine. These procedures are not without costs or complications. These findings, along with information about the characteristics of the diagnosed cancers, many of which are slow-growing and have an indolent course, have led many to conclude that we are in the midst of an epidemic of thyroid cancer overdiagnosis and overtreatment [1-3]. We present a case in which the pursuit of a diagnosis in an individual with a suspicious thyroid nodule led to significant harms.

## Case presentation

A 66-year-old Caucasian man with metastatic melanoma and unresectable, locally advanced pancreatic adenocarcinoma was admitted to the intensive care unit (ICU) with an anaphylactoid reaction following the attempted biopsy of a thyroid nodule.

The melanoma had initially been diagnosed by shave biopsy of the back in September 2015. The patient underwent wide excision in October 2015. He then developed painful axillary lymphadenopathy, which a fine-needle aspiration biopsy (FNAB) in March 2016 confirmed was recurrent, metastatic melanoma with the BRAF V600E mutation. His pancreatic malignancy was initially discovered during staging for melanoma on axial imaging of the chest, abdomen, and pelvis in October 2015. A large, low-density infiltrative pancreatic mass was demonstrated on computed tomography (CT), along with tumor involvement of the splenic and hepatic arteries and portal, superior mesenteric, and splenic veins. The diagnosis of pancreatic adenocarcinoma was confirmed by sampling via endoscopic ultrasound. He had received two cycles of chemotherapy with gemcitabine and protein-bound paclitaxel, though this had been temporarily stopped due to gastrointestinal side effects. A hereditary disorder such as melanoma-pancreatic cancer syndrome was postulated, although not confirmed by genetic testing.

**Figure 1:**
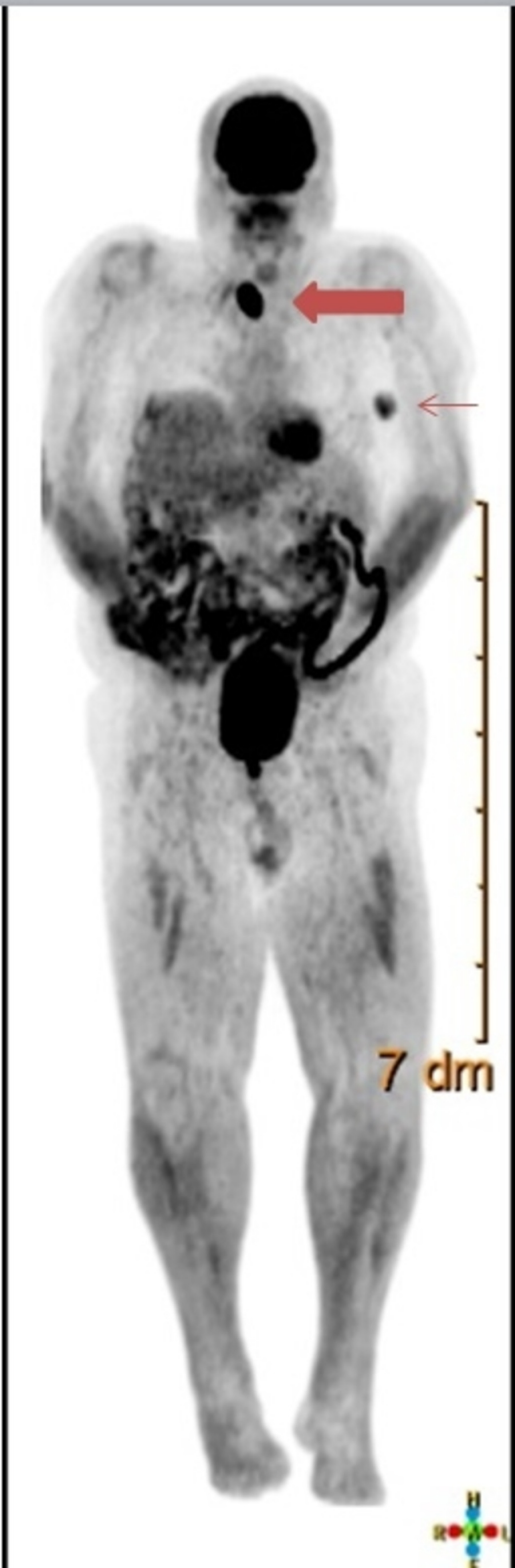
PET/CT scan The right thyroid gland (thicker arrow) is enlarged with increased metabolic activity with a maximum SUV of 14.4, which is suspicious for malignancy. The left axillary lymph node (thinner arrow) is enlarged with a maximum SUV of 4.3, which is concerning for melanoma involvement.

During a re-staging positron emission tomography (PET) scan in April 2016, increased metabolic activity with maximum standardized uptake value (SUV) of 14 was seen in the right thyroid, corresponding to a moderately hypervascular nodule measuring 3.1 x 2.8 x 3.8 cm by ultrasound (Figure 1). This was suspicious for malignancy. At this time, the patient had normal thyroid function tests and was clinically euthyroid. 

The patient opted to undergo further diagnostic evaluation of the thyroid nodule by fine needle aspiration biopsy (FNAB) in August 2016. Following tissue infiltration with 1% lidocaine to the anterior neck at the beginning of the procedure, the patient became tachycardic and hypotensive. The procedure was aborted and the patient was transferred to the ICU.

Upon arrival to the ICU, he was afebrile with an initial heart rate of 131 beats per minute, blood pressure 66/42 mmHg, and saturating 98% on room air. He complained only of abdominal discomfort and new diarrhea. He denied knowledge of an allergy to lidocaine. His exam was notable for an older man in no apparent distress, with non-tender and benign abdominal exam. His initial laboratory evaluations were notable for leukocytosis (white blood cell count of 18.6 x 109/L), and serum lactic acid of 2.4 mmol/L. The patient received intravenous fluids with improvement in his tachycardia and hypotension. Further infectious evaluations were negative, including blood cultures, urine culture, stool culture, stool ova and parasite, C. difficile toxin polymerase chain reaction (PCR), and chest x-ray. Laboratory evaluations on the second day of admission demonstrated normalization of his WBC count, but revealed evidence of acute kidney injury (serum creatinine 2.2 mg/dL, with a prior normal baseline), with the latter attributed to prerenal azotemia from his hypotensive episode. The patient did not require antibiotics, nor did he require vasoactive agents. He was discharged home on hospital day two after improvement and stabilization in his clinical status. On repeat laboratory assessment two weeks later, his renal function had normalized.

Upon follow up with his oncologist, he was started on immunotherapy for melanoma with nivolumab after discussions of the risks and benefits. Further evaluation of the thyroid nodule was not pursued.

## Discussion

Overdiagnosis occurs when testing modalities identify asymptomatic cancers that are unlikely to be clinically meaningful during a patient’s lifetime. An increase in medical access, added emphasis on surveillance, and an improvement in the sensitivity and specificity of imaging modalities have contributed to the overdiagnosis of thyroid malignancies. Many of these cancers are small, localized, asymptomatic, and represent indolent cancers. Overtreatment refers to management options whose benefits do not outweigh the risks. Surgical and radiation options for indolent thyroid cancers contribute to increased financial and psychological burdens on the patient, as well as increasing societal costs [3]. Indeed, in this patient with locally invasive pancreatic cancer and malignant melanoma, there was a very low likelihood that diagnosing and treating an incidental thyroid cancer would be beneficial.

The 2015 American Thyroid Association (ATA) guidelines for the diagnosis and management of thyroid nodules and thyroid cancer strongly recommend FNAB sampling of a nodule with increased focal uptake on a PET scan corresponding to an ultrasound-confirmed nodule greater than 1 cm in size or with a suspicious sonographic pattern. Suspicious findings on ultrasound of the neck include the presence of microcalcifications, irregular margins, hypoechogenicity, and solid nodules [4].

The ATA guidelines also note that about a third of PET-avid nodules are cancerous, but that in a patient with known non-thyroidal cancer, a nodule more often represents papillary thyroid carcinoma rather than a metastatic lesion [5]. There are no guidelines regarding the diagnostic studies recommended for thyroid nodules in individuals with known non-thyroidal malignancies. The prevalence of thyroid nodules incidentally detected during evaluation for metastatic disease in individuals with existing malignancies was 2.2% in one study [6]. The most frequent primary malignancies which metastasize to the thyroid include renal cell carcinoma, colorectal cancer, and cancers of the lung and breast; the malignancies that our patient had can infrequently metastasize to the thyroid. Diagnostic yield of FNAB in such cases may be problematic and often requires additional immunohistochemical staining for confirmation of tumor origin [5].

FNAB of the thyroid is a relatively safe procedure. Its main limitation is that it often results in an inadequate specimen or nondiagnostic sample. The main complication of thyroid FNAB is pain, which may be managed by the use of a local anesthetic, although this is not strictly necessary [7].

Although usually considered a benign medication, lidocaine is one of the most common causes of local anesthetic systemic toxicity (LAST). A literature review of LAST found 67 cases reported between 2010 and 2014, with 33% of these due to lidocaine. LAST may occur with local anesthetic injection into peripheral nerves, as well as into the epidural space. Isolated neurological signs and symptoms are the most common clinical presentation of LAST, occurring in 50% of cases, and may include seizures. Isolated cardiovascular symptoms, as in our patient, occur in 14% of cases and encompass both tachy- and bradycardia as well as hypo- and hypertension [8]. The likelihood of cardiovascular toxicity is known to be higher in less potent anesthetics like lidocaine [9]. In our patient following the administration of lidocaine, LAST was limited to cardiovascular symptoms, which resolved quickly with supportive care. In cases with more severe cardiovascular symptoms, epinephrine may be used. Lipid emulsion therapy has also been used in severe cases [9]. The overall mortality is 10% [8]. LAST may recur with subsequent exposures to the anesthetic, as described in a case report [10]. 

## Conclusions

Overtreatment of thyroid malignancies, particularly those likely to be indolent, may result in risks that outweigh the benefits of treatment. These risks include the complications of surgery, as well as the costs incurred and the burden on quality of life. Our patient experienced an adverse reaction while undergoing diagnostic evaluation for suspicion of a thyroid cancer in the setting of two other lethal primary malignancies. The adverse reaction precipitated by the attempted FNAB resulted in not only harm and discomfort to the patient, but also in a costly stay in the ICU. It is as important to address goals of diagnostic testing as it is for treatment, particularly if further studies are unlikely to change overall management or outcomes.
